# The deubiquitinase function of ataxin-3 and its role in the pathogenesis of Machado-Joseph disease and other diseases

**DOI:** 10.1042/BCJ20240017

**Published:** 2024-03-18

**Authors:** Anastasiya Potapenko, Jennilee M. Davidson, Albert Lee, Angela S. Laird

**Affiliations:** Motor Neuron Disease Research Centre, Macquarie Medical School, Faculty of Medicine, Health and Human Sciences, Macquarie University, Sydney, NSW 2109, Australia

**Keywords:** deubiquitinase, neurodegeneration, polyglutamine repeat, spinocerebellar ataxia type-3, ubiquitin proteasome system

## Abstract

Machado-Joseph disease (MJD) is a devastating and incurable neurodegenerative disease characterised by progressive ataxia, difficulty speaking and swallowing. Consequently, affected individuals ultimately become wheelchair dependent, require constant care, and face a shortened life expectancy. The monogenic cause of MJD is expansion of a trinucleotide (CAG) repeat region within the *ATXN3* gene, which results in polyglutamine (polyQ) expansion within the resultant ataxin-3 protein. While it is well established that the ataxin-3 protein functions as a deubiquitinating (DUB) enzyme and is therefore critically involved in proteostasis, several unanswered questions remain regarding the impact of polyQ expansion in ataxin-3 on its DUB function. Here we review the current literature surrounding ataxin-3's DUB function, its DUB targets, and what is known regarding the impact of polyQ expansion on ataxin-3's DUB function. We also consider the potential neuroprotective effects of ataxin-3's DUB function, and the intersection of ataxin-3's role as a DUB enzyme and regulator of gene transcription. Ataxin-3 is the principal pathogenic protein in MJD and also appears to be involved in cancer. As aberrant deubiquitination has been linked to both neurodegeneration and cancer, a comprehensive understanding of ataxin-3's DUB function is important for elucidating potential therapeutic targets in these complex conditions. In this review, we aim to consolidate knowledge of ataxin-3 as a DUB and unveil areas for future research to aid therapeutic targeting of ataxin-3's DUB function for the treatment of MJD and other diseases.

## Introduction

The human *ATXN3* gene is located on chromosome 14q32.1 [1] and encodes the ubiquitously expressed [2–6], and evolutionarily conserved [7,8] ataxin-3 protein (UniProt ID P54252). Expansion of the cytosine-adenine-guanine (CAG) repeat in exon 10 of the *ATXN3* gene [9] results in expansion of the polyglutamine (polyQ) repeat region of the ataxin-3 protein. Such expansion is causative for the neurodegenerative disease Machado-Joseph disease (MJD) [1], also known as spinocerebellar ataxia type 3. For this reason, studies of the ataxin-3 protein have predominantly focused on unravelling its pathogenic role in MJD. However, aside from its involvement in MJD, the ataxin-3 protein is intimately involved in an eclectic range of biological processes ranging from protein quality control [10,11], autophagy [12], transcription regulation [13,14], cytoskeletal regulation [15,16], stress responses [17], and antiviral response [18], to DNA repair [19]. Ataxin-3 has also been implicated in the progression of numerous cancers including testicular [20], breast [21], gastric [22], and thyroid cancers [23].

Structurally, ataxin-3 is 42 kDa in size and contains a deubiquitinating (DUB) Josephin domain within its N-terminus, a ubiquitin-binding domain within its C-terminus, and a polyQ repeat region of variable size. The function of ataxin-3 as a DUB enzyme is an emerging area of interest given that aberrant deubiquitination has been linked to both neurodegeneration [24,25], and many types of tumours [26]. Ataxin-3's DUB function also appears to be capable of regulating transcription.

## Machado-Joseph disease

MJD is an incurable neurodegenerative disease characterised by the development of ataxia, muscle weakness, dysarthria, and vision changes [27–29]. After symptom onset, the survival of individuals with MJD is ∼20–25 years, with later stages of the disease culminating in wheelchair dependence [30,31]. Whilst spinocerebellar ataxias are considered rare diseases, MJD is the most common inherited form of spinocerebellar ataxia globally [32].

MJD belongs to a family of inherited neurodegenerative diseases termed ‘polyQ expansion diseases’ [33]. Each disease within this family is characterised by a genetic mutation resulting in expansion of the polyQ region in the respective disease protein [34]. In the context of MJD, expansion of the trinucleotide CAG [9] occurs due to mutation of the *ATXN3* gene on chromosome 14 [1]. While healthy individuals carry 12–40 CAG repeats, individuals with MJD typically carry 62–84 repeats [35,36], sometimes more, and the size of the CAG repeat is directly proportional to the severity of MJD symptoms, and inversely proportional to the age of disease onset [35,37–39]. The consequence of CAG repeat expansion in the *ATXN3* gene is polyQ expansion within the ataxin-3 protein. PolyQ-expanded ataxin-3 has a propensity to misfold, aggregate, and form intranuclear inclusions [4,40–43]. Neurodegeneration is widespread in MJD, mainly involving atrophy of the cerebellum, brainstem, and basal ganglia, although there is no definitive correlation between the location of intranuclear inclusions and the pattern of neurodegeneration [28]. Interestingly, intranuclear inclusions observed in MJD brains contain ataxin-3, as well as ubiquitin and proteasomal subunits [44,45], suggesting that mutant ataxin-3 is insufficiently cleared [46]. Ubiquitin-positive inclusions were established to be a shared feature of polyQ diseases over two decades ago [47], and they are also observed in other neurodegenerative diseases including Alzheimer's disease, amyotrophic lateral sclerosis (ALS), Parkinson's disease, and prion diseases [48,49]. Taken together, these studies suggest that impairment of the ubiquitin-proteasome system (UPS) is a central link between neurodegenerative diseases featuring protein misfolding and aggregation [50,51]. Although the function of ataxin-3 as a DUB enzyme within the UPS and changes in this function due to polyQ expansion have been studied as reviewed below, many aspects remain to be explored.

## The ubiquitin-proteasome system

Neurodegenerative diseases such as spinocerebellar ataxias (including MJD), Parkinson's disease, and Huntington's disease have been associated with dysfunction of the UPS [52]. Ataxin-3 plays a key role in sequestering proteins to proteasomal degradation [11] and is therefore closely involved in protein quality control [28,53]. Both wild-type and polyQ-expanded ataxin-3 are also degraded by the UPS [54].

## The ubiquitination cascade

Ubiquitination is a post-translational modification controlling protein degradation, protein-protein interactions, and the localisation of proteins within the cell [55]. The attachment of ubiquitin to target proteins sequesters them to diverse cellular pathways, including degradation via the UPS [52]. Ubiquitin is a protein that reversibly binds to target proteins via an isopeptide linkage of its C-terminal carboxyl group, typically to lysine (K) residues within the target protein [56]. The process of ubiquitination is mediated by ubiquitin-activating enzymes (E1), ubiquitin-conjugating enzymes (E2), and ubiquitin ligases (E3) [57]. Ubiquitination can occur at a single K residue (monoubiquitination), or at several K residues (multiubiquitination) [58,59], and additional cycles of ubiquitination can result in a chain of ubiquitin forming on a single K residue (polyubiquitination) [59,60]. Assembly of ubiquitin chains on a target protein requires formation of a covalent linkage between the C-terminus of ubiquitin and the ε-amino group of any of the seven K residues within ubiquitin (K6, K11, K27, K33, K48, and K63), or to the α-amino group of the methionine (M1) residue [61]. Linear and branched chains containing a combination of multiple ubiquitin linkage types and ubiquitin-like proteins have also been identified in cells [62–65]. Examples of ubiquitin-like proteins include small ubiquitin-like modifier (SUMO) and neural precursor cell-expressed developmentally down-regulated protein 8 (NEDD8). Protein ubiquitination of non-lysine residues such as cysteine, serine, and threonine, as well as the N-terminal amino group of proteins, can also contribute to various cellular processes (reviewed in [66]).

## Canonical lysine ubiquitin linkages on proteins

The nature of ubiquitin chains attached to a target protein determines the protein's fate [67]. Ubiquitin linkages occurring between the C-terminus of one ubiquitin and lysine at position 48 (K48) of the previous ubiquitin [68], are the most common linkage type targeting proteins for proteasomal degradation [69,70]. K63 linkages are the next most common linkage type [71] and are found to sequester proteins to pathways including transcriptional activation, DNA damage repair, and autophagy [72,73]. K63-linked ubiquitin chains are also thought to play a role in the biogenesis of protein inclusions [73–75]. In addition to being the most abundant ubiquitin chain type found in cells [76], K48-linked ubiquitin chains are also the most prevalent ubiquitin chain type found on the ataxin-3 protein [77].

## Ataxin-3 functions as a DUB enzyme

DUB enzymes cleave and regulate multiple types of ubiquitin chains from target substrates, therefore rescuing them from degradation [78], and regulating their stability [79,80]. DUB enzymes are emerging as critical regulators in cancer (reviewed in [81]) and neurodegenerative diseases, with their dysfunction linked to Alzheimer's, Parkinson's, and Huntington's diseases (reviewed in [24,82]). Ataxin-3 is a well established ubiquitin-specific protease [83–86], and it is known to function as a DUB enzyme within the UPS [10,83,87–91].

**Figure 1.**
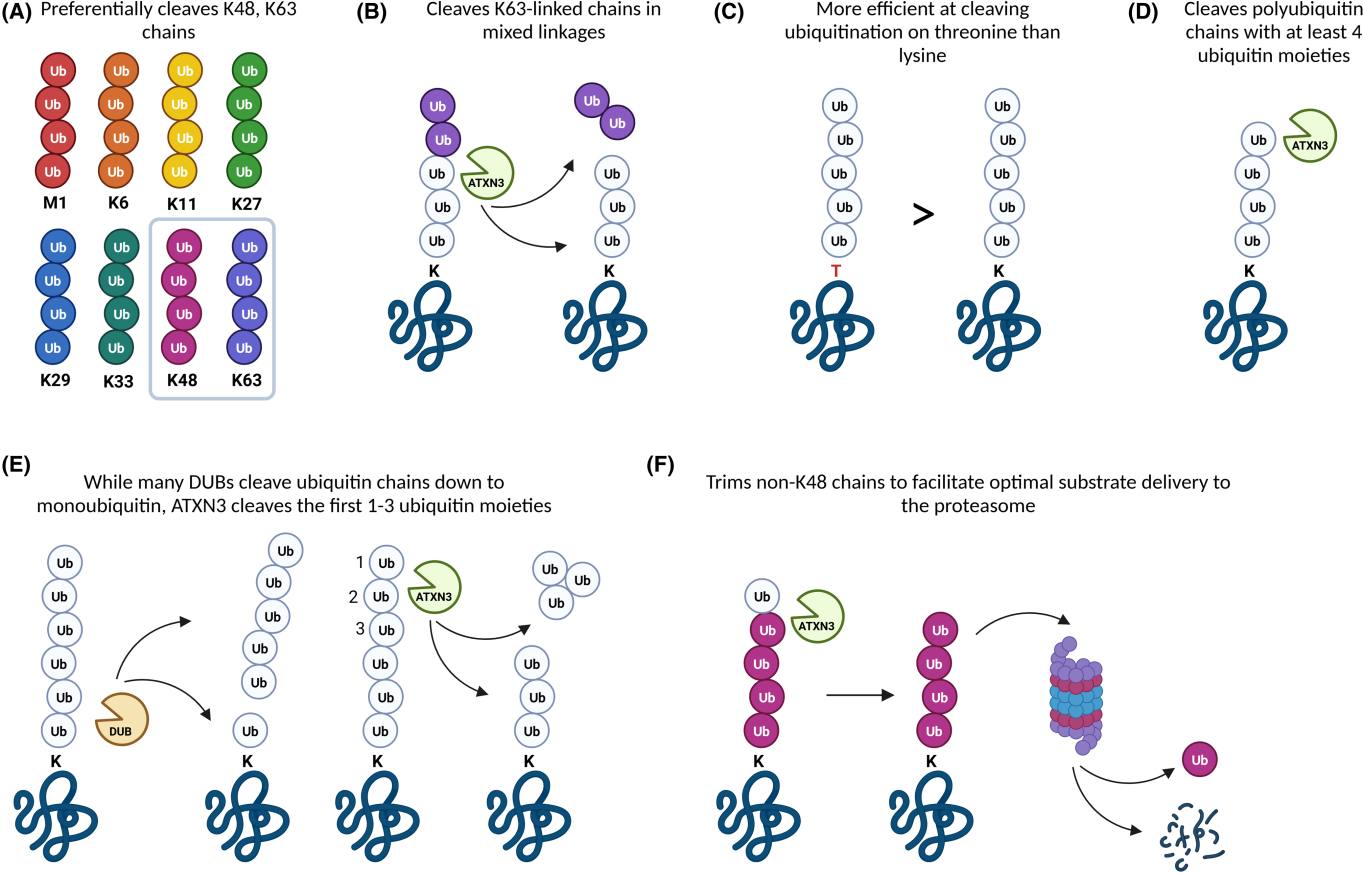
As a deubiquitinating enzyme in the ubiquitin-proteasome system, ataxin-3 functions in many ways. (**A**) Preferentially cleaves K48- and K63-linked lysine ubiquitination. (**B**) Preferentially cleaves K63-linked ubiquitin chains within mixed linkage chains. (**C**) Is more efficient at cleaving ubiquitination on threonine than lysine residues. (**D**) Preferentially cleaves polyubiquitin chains comprised of at least four ubiquitin moieties and is inefficient at cleaving polyubiquitin chains of less than six ubiquitin moieties. (**E**) Functions as a chain-editing DUB that cleaves the first 1–3 ubiquitin moieties from a polyubiquitin chain and is inefficient at cleaving subsequent ubiquitin moieties. (**F**) Is proposed to trim non-K48-linked ubiquitin linkages to facilitate optimal delivery of substrates to the proteasome. K is lysine, T is threonine, DUB is deubiquitinating enzyme. Figure created with BioRender.com.

The finding of increased levels of ubiquitinated proteins in *Atxn3* knockout mice provided the first *in vivo* evidence of ataxin-3's DUB role [8]. Ubiquitinated proteins were similarly shown to accumulate in cells expressing catalytically inactive ataxin-3 protein [92], providing further evidence in support of ataxin-3 functioning as a DUB enzyme. Within its role as a DUB, ataxin-3 is capable of binding both K48- and K63-linked ubiquitin chains, however, preferentially cleaves K63-linked chains within mixed linkages, as well as very long or possibly structurally aberrant ubiquitin chains (Figure 1) [88]. Ataxin-3 preferentially cleaves polyubiquitin chains containing at least four ubiquitin moieties [88], which is the minimum chain length required for sequestration of proteins for proteasomal degradation [93], and is inefficient at cleaving chains with fewer than six ubiquitin moieties [7]. As the first identified DUB capable of cleaving mixed linkage ubiquitin chains, ataxin-3 is hypothesised to prevent the accumulation of large and aberrantly structured ubiquitin chains within cells [88]. While some DUB enzymes completely dismantle polyubiquitin chains down to monoubiquitin [94], both full-length ataxin-3 and the isolated ataxin-3 Josephin domain, instead seem to cleave the first one, two, or three ubiquitin moieties from a chain, and are less efficient at cleaving any subsequent ubiquitin moieties [88,95]. Ataxin-3 is therefore proposed to function as a chain-editing DUB enzyme, which may edit ubiquitin chains as they are produced, and can possibly trim non-K48-linked ubiquitin linkages to facilitate optimal delivery of substrates to the proteasome [83,88,95–97]. Ataxin-3 is also capable of cleaving other ubiquitin linkage types and was shown to have higher esterase activity toward serine over threonine peptides, and is more efficient at cleaving ubiquitin attached to threonine than lysine residues [98].

**Figure 2.**
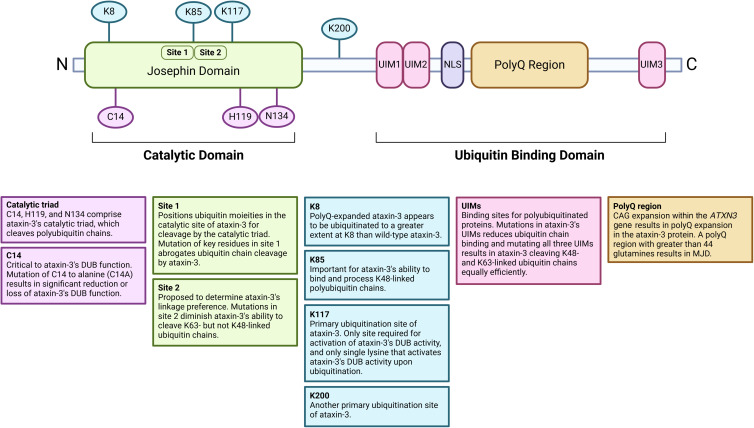
Schematic representation of ataxin-3's structure. The N-terminus of ataxin-3 contains the deubiquitinating Josephin domain within which two ubiquitin-binding sites and the polyubiquitin chain cleaving catalytic triad are located. The C-terminus of ataxin-3 represents the ubiquitin binding domain, which contains the three ubiquitin-interacting motifs (UIMs), a nuclear localisation signal (NLS), and a polyQ repeat which is expanded in MJD. Important lysine ubiquitination sites on ataxin-3 are indicated in blue. Figure created with BioRender.com.

The human ataxin-3 protein is ubiquitously expressed and appears to have evolutionarily conserved functions [99]. Ataxin-3 contains a catalytic Josephin domain at its N-terminus, tandem ubiquitin-interacting motifs (UIMs) at its C-terminus, and a polyQ repeat region of variable size (Figure 2). PolyQ expansion does not significantly alter ataxin-3's polyubiquitin binding capacity *in vitro* [84], however, polyQ-expanded ataxin-3 appears to be less efficient than its wild-type counterpart at reducing ubiquitinated proteins in cells, which indicates that expansion may somehow impair its DUB function [88]. Further suggestion of altered DUB function of polyQ-expanded ataxin-3 stems from a recent study reporting that the amount of K48- and K63-ubiquitinated proteins varies between brain regions and with disease stage within the brain of MJD mice [100].

Ataxin-3's Josephin domain hosts the catalytic triad comprised of cysteine 14 (C14), histidine 119 (H119), and asparagine 134 (N134) residues [83,101], which marks the active site of the protein. When C14 is mutated to alanine (C14A), there is significant reduction [83] or loss [10,96] of ataxin-3's DUB function, implicating C14 as a critical residue for ataxin-3's DUB function. The Josephin domain also encompasses two ubiquitin binding sites — site 1 and site 2 [102]. Site 1, which lies adjacent to the C14 residue, appears to anchor ubiquitin moieties and correctly position them into the active site of ataxin-3 for cleavage by the catalytic triad [95]. Site 1 is critical to ataxin-3's DUB role given that mutation of key residues within this site abrogates ubiquitin chain cleavage by the isolated Josephin domain as well as by full-length ataxin-3 [95]. Site 2 seems comparatively less foundational to ataxin-3's DUB role since mutation of the site does not markedly modify the cleavage activity of the Josephin domain [95]. Rather, site 2 is proposed to play a role in determining ataxin-3's linkage preference, given that mutations within this site diminish ataxin-3's ability to cleave K63- but not K48-linked ubiquitin chains [95]. The Josephin domain also contains many sites of lysine ubiquitination, including the K117 residue, which when ubiquitinated, dramatically enhances the DUB activity of ataxin-3 [90,103] (Figure 2).

The UIMs are sites where mono and polyubiquitinated proteins bind [104–106] for subsequent cleavage by the catalytic triad. Together, the tandem UIMs of ataxin-3 form the third ubiquitin binding site within the protein [92]. Wild-type ataxin-3 preferentially cleaves K63-linked ubiquitin chains [88]. However, mutating key residues within ataxin-3's UIMs reduces ubiquitin chain binding [10,83,84,92] and mutating all three UIMs results in ataxin-3 cleaving K48- and K63-linked chains equally efficiently [88]. Ataxin-3's UIMs therefore appear to be critical to its DUB function. In support of this, the K85 residue (located between ubiquitin-binding sites 1 and 2) is important for ataxin-3's binding and processing of K48-linked polyubiquitin chains [107] through the previously described mechanism involving the UIMs and site 2 [108]. K85 is also important for cytoplasmic localisation of ataxin-3 [107].

The most extensively studied isoforms of ataxin-3 protein are ataxin-3a (UniProt ID P54252-1, also known as MJD1a) and ataxin-3c (UniProt ID P54252-2), which are created by alternate splicing and differ mainly in their C-terminal regions [9,109,110]. Ataxin-3c is the principal isoform expressed in human and rodent brain tissue [111], although both isoforms are expressed in the brain and are known to bind ubiquitinated substrates [92]. Some isoforms of ataxin-3, such as ataxin-3c, host a third UIM which appears to be dispensable for ataxin-3's ubiquitin-binding capacity [84,112].

Ataxin-3 is also involved in other degradation pathways, such as endoplasmic reticulum-associated protein degradation (ERAD), which degrades incorrectly folded proteins [113–115]. Valosin-containing protein (VCP/p97) is the cornerstone protein in ERAD. VCP/p97 (and cofactors) binds ubiquitinated substrates and assists in their retro translocation from the endoplasmic reticulum into the cytosol for proteasomal degradation [116,117]. However, when ataxin-3 binds to VCP/p97, the interaction between VCP/p97 and its cofactor, ubiquitin fusion degradation protein 1 (Ufd1), is decreased, consequently leading to decreased interaction of VCP/p97 with ubiquitinated proteins. This results in decreased retro translocation of ubiquitinated ERAD substrates to the cytosol, causing an accumulation of proteins in the endoplasmic reticulum [11]. Ataxin-3 and VCP/p97 co-interact independently of ataxin-3's DUB activity and its UIM domains [11]. Moreover, expression of catalytically inactive C14A mutant and UIM mutant forms of ataxin-3 decrease the binding of ubiquitinated proteins to VCP/p97, suggesting that the decreased binding of ubiquitinated proteins to VCP/p97 is not due to ataxin-3 cleaving or binding to ubiquitin chains [11]. Since these accumulated ERAD substrates are polyubiquitinated and sequestered to VCP/p97, it has been proposed that ataxin-3-mediated deubiquitination of ERAD substrates may be coupled to their transfer from VCP/p97 to the proteasome [118].

VCP/p97 also influences ataxin-3's DUB activity. VCP/p97 activates the DUB activity of wild-type, but not polyQ-expanded ataxin-3, despite interacting more with polyQ-expanded ataxin-3 [11]. It is possible that the increased co-interaction decreases substrate retro translocation from the endoplasmic reticulum [11,119]. It is also possible that without activation by VCP/p97, polyQ-expanded ataxin-3 may function less efficiently as a DUB, resulting in accumulation of ubiquitinated proteins in cells [120].

## Ubiquitination of ataxin-3 may regulate its DUB function

The regulation of a DUB's enzymatic activity by ubiquitination is thought to be a common regulatory mechanism amongst DUB enzymes [121]. Ataxin-3 itself is ubiquitinated, and both wild-type and polyQ-expanded ataxin-3 appear to be ubiquitinated to similar extents [122]. The pattern of ubiquitin linkages on both forms of ataxin-3 are also reportedly similar, suggesting that toxicity and aggregation of polyQ-expanded ataxin-3 is unlikely to be caused by differences in ubiquitin linkage types [77].

The primary ubiquitination sites of both wild-type and polyQ-expanded ataxin-3 are the K117 and K200 lysine residues [77], with the K117 residue being the only site required for activation of ataxtin-3's DUB activity, and the only single lysine that was found to activate ataxin-3's DUB activity upon ubiquitination [90]. However, the mechanism by which ubiquitination of ataxin-3 at K117 regulates the DUB function of ataxin-3, is yet to be established [77].

Compared with its wild-type counterpart, polyQ-expanded ataxin-3 appears to be ubiquitinated to a greater extent at the K8 lysine residue [77]. The K8 and K85 residues appear to be important for the proteostasis of ataxin-3 as the presence of either residue within ataxin-3 increases the stability of the protein compared with ataxin-3 with all lysine residues mutated to arginine [107]. As the K8 residue is located in close proximity to the critical C14 residue of the catalytic Josephin domain, it has also been suggested that enhanced ubiquitination at this site may impact polyQ-expanded ataxin-3's DUB function [77]. Ubiquitination of ataxin-3 at the K8 residue has also been suggested by a recent study [107].

Ubiquitination of ataxin-3 does not appear to impact its subcellular localisation [92] or alter its preference for cleaving K63 ubiquitin linkages [90]. Rather, ubiquitination of ataxin-3 appears to regulate its activity during cellular stress because impairment of the proteasome, increases in cellular abundance of ubiquitin, and induction of the unfolded protein response all increase the ubiquitination of ataxin-3 [90]. The ubiquitination of ataxin-3 also appears to be dependent on its UIMs [92], which is consistent with the UIM domains within other proteins mediating their ubiquitination [121,123,124]. Whether ataxin-3 has intrinsic DUB activity toward other ataxin-3 polypeptides is somewhat contested with some studies suggesting it is possible [92], and other studies suggesting that ataxin-3 does not function in this way [90].

## Comparison of the MJD DUB family

The MJD family of DUBs (reviewed by [125]) is the smallest of the DUB families and contains four members: ataxin-3, ataxin-3-like protein (ataxin-3L), and Josephin domain-containing proteins 1 and 2 (JosD1, JosD2) [126]. These four proteins are unified by the presence of a catalytic Josephin domain of ∼180 amino acids, though ataxin-3 is the only MJD DUB implicated in the pathophysiology of MJD. Catalytic residues within the Josephin domain are relatively conserved amongst the four MJD DUBs [127], and while ataxin-3 and ataxin-3L also share the UIMs and a C-terminal polyQ domain, the JosD1 and JosD2 proteins are essentially comprised solely of a Josephin domain [126]. Despite ataxin-3 and ataxin-3L proteins possessing high sequence homology of their DUB Josephin domain (85%), ataxin-3L has considerably higher DUB activity and is in fact the most active of the four MJD DUBs [128]. The four MJD DUBs are also functionally distinct. The biological functions of ataxin-3L are largely undetermined, however its DUB function has been linked to the progression of breast cancer [129]. JosD1 is predominantly localised to the plasma membrane where it appears to regulate membrane dynamics, cell motility, and endocytosis [127]. JosD2 is primarily found in the cytoplasm [127] and is hypothesised to play a role in protein quality control given that it cleaves K11, K48, and K63 ubiquitin linkages [130], all three of which are implicated in protein degradation [131]. Interestingly, the DUB activity of JosD1 is only activated upon monoubiquitination [127], which is reminiscent of ubiquitination of ataxin-3 increasing its catalytic activity [90,103] and perhaps suggests that regulation of a DUB's activity by ubiquitination may be a more common regulatory mechanism than previously considered [127].

## Identified substrates of ataxin-3's DUB function

Several substrates of ataxin-3's DUB activity have been identified. The nature of their interaction with ataxin-3 and the impact of polyQ expansion on these interactions is summarised in Table 1. Identifying additional substrates of ataxin-3's DUB function and profiling how their interaction with ataxin-3 is impacted by polyQ expansion in ataxin-3 would be valuable for the development of novel targets for therapeutic treatment of MJD and other complex diseases [137].

**Table 1.** Summary of the known substrates of ataxin-3's deubiquitinating function, with key references

Substrate	Known functions	Normal interaction with ataxin-3	Impact of polyQ expansion	Key references
Carboxyl terminus of Hsp70-interacting protein (CHIP)	E3 ubiquitin ligase	Ataxin-3 trims polyubiquitin chains on CHIP to modulate CHIP activity in the ubiquitinating complex.	Binding affinity is greater for polyQ-expanded ataxin-3 and CHIP. PolyQ-expanded ataxin-3 results in decreased levels of CHIP in the brain.	[132–134]
Parkin	E3 ubiquitin ligase	Ataxin-3 cleaves self-ubiquitination chains on parkin, regulating parkin activity but not parkin abundance. Parkin ubiquitinates and facilitates clearance of polyQ-expanded ataxin-3 fragments.	PolyQ-expanded ataxin-3 more efficiently cleaves K27- and K29-linked ubiquitin chains on parkin. Removal by polyQ-expanded ataxin-3 may result in enhanced degradation of parkin by autophagy. Parkin levels are significantly reduced in MJD mice and cells.	[89,135,136]
Tumor protein p53 (p53)	Tumour suppressor protein and regulator of apoptosis	Ataxin-3 deubiquitinates p53 preventing its degradation by the UPS and thereby stabilising p53.	PolyQ-expanded ataxin-3 stabilises p53 and enhances p53-mediated apoptosis. Levels of p53 are elevated in transgenic MJD mice, and aberrant activation of the p53 pathway has been reported in MJD patient brains and MJD models.	[137–142]
Checkpoint kinase 1 (Chk1)	Regulator of the cell cycle and DNA damage response	Ataxin-3 deubiquitinates Chk1, protecting it from proteasomal degradation, and therefore promoting DNA repair and checkpoint signalling.	PolyQ expansion does not appear to alter ataxin-3's capacity to deubiquitinate and therefore stabilise Chk1.	[143]
Mediator of DNA damage checkpoint protein 1 (MDC1)	Regulator of cell cycle and recruiter of repair proteins to DNA damage sites	Ataxin-3 deubiquitinates MDC1, preventing its premature removal, which promotes MDC1-mediated double-stranded DNA break repair.	Unknown.	[19]
Beclin 1	Key regulator of autophagy and apoptosis	Ataxin-3 deubiquitinates beclin 1, rescuing beclin 1 from proteasomal degradation.	PolyQ-expanded ataxin-3 binds with higher affinity to beclin 1 and deubiquitinates it less, resulting in increased proteasomal degradation of beclin 1. PolyQ-expanded ataxin-3 increases K48-linked ubiquitination of beclin 1. Reduced beclin 1 abundance has been reported in rodent MJD models and MJD patient brains.	[12,144–146]
Late Endosomal/Lysosomal Adaptor, MAPK And MTOR Activator 1 (LAMTOR1)	Part of Ragulator complex, involved in activating mammalian target of rapamycin complex 1 (mTORC1)	Ataxin-3 is phosphorylated by NOD2 (nucleotide-binding oligomerisation domain-containing protein 2) and TLR2 (toll-like receptor 2) and this results in ataxin-3 deubiquitinating LAMTOR1.	Unknown.	[147]
CREB-binding protein (CBP)	Transcriptional co-activator, forms complexes that result in histone acetylation	Knockdown of ataxin-3 results in enhanced polyubiquitination of CBP, suggesting ataxin-3 may deubiquitinate CBP.	Unknown.	[148]
Hypoxia-inducible factor 1-alpha (HIF1α)	Key transcriptional regulator, hypoxia signalling	Ataxin-3 deubiquitinates HIF1α. Depletion of ataxin-3 results in a significant increase in HIF1α in cells.	Unknown.	[147]
Phospholipase D3 (PLD3)	Hydrolysis of phospholipids	Ataxin-3 deubiquitinates PLD3.	Unknown.	[147]
Krüppel-like factor 4 (KLF4)	Transcription factor	Ataxin-3 deubiquitinates KLF4, stabilising the protein, and promoting breast cancer metastasis.	Unknown.	[21]
Yes-associated protein (YAP)	Transcriptional co-activator and effector protein in Hippo signalling pathway	Ataxin-3 removes K48-linked chains from YAP, stabilising YAP and thus promoting prostate cancer progression.	Unknown.	[149]
Histone deacetylase 3 (HDAC3)	Regulates gene expression	Ataxin-3 removes K48- and K63-linked polyubiquitin chains on HDAC3.	Unknown.	[14,18]
Voltage-dependent anion channel 1 (VDAC1)	Outer mitochondrial membrane protein	Ataxin-3 can deubiquitinate polyubiquitinated VDAC1 and is therefore important for parkin-VDAC1-mediated mitophagy.	Deubiquitination of VDAC1 is stronger in MJD patient-derived fibroblasts than controls.	[150]

PolyQ, polyglutamine.

## What is the impact of polyglutamine expansion on ataxin-3's DUB function?

While a growing body of evidence suggests that polyQ expansion in ataxin-3 may impact its DUB function [88,100], whether this results in a gain or loss of DUB function and the impact of such changes on ataxin-3's targets is yet to be comprehensively evaluated, as detailed in Table 1. The broader implications of changes in ataxin-3's DUB function due to polyQ expansion in the context of MJD pathophysiology also remain to be clarified.

One example of how ataxin-3's DUB function is impacted by polyQ expansion is its relationship with the E3 ubiquitin ligase parkin. PolyQ expansion has been suggested to result in a gain-of-function effect culminating in enhanced degradation of parkin. Ataxin-3 deubiquitinates parkin in cells and in recombinant *in vitro* systems, in a manner that involves its UIMs [89]. PolyQ expansion does not alter binding between ataxin-3 and parkin, however, it enhances ataxin-3's deubiquitination of parkin, particularly by cleaving K27- and K29-linked ubiquitin chains [89]. Parkin ubiquitinates itself predominantly via K27- and K29-linked chains which may protect it from autophagic degradation, and preferential cleavage of these chains by polyQ-expanded ataxin-3 can result in enhanced autophagic degradation of parkin, as reflected by a profound reduction in parkin levels in MJD mice [89]. As such, it is hypothesised that the Parkinsonism features observed in some patients with MJD may result from polyQ-expanded ataxin-3-mediated degradation of parkin as a consequence of this gain-of-function effect. An alternative explanation for the enhanced clearance of parkin observed in MJD is that the complex formed by the interaction of polyQ-expanded ataxin-3 and parkin may result in the autophagic degradation of parkin simultaneously [151]. It is therefore unclear whether enhanced clearance of parkin represents a gain of polyQ expanded ataxin-3's DUB function or is merely a direct consequence of the expanded polyQ region.

Curiously, expansion of the polyQ region of ataxin-3 appears to result in the targeted destruction of another E3 ubiquitin ligase — carboxyl terminus of Hsp70-interacting protein (CHIP). Monoubiquitination of CHIP by Ube2w, an initiator E2, promotes recruitment of ataxin-3 to the CHIP ubiquitination complex before ubiquitin chains are assembled on substrates of CHIP [132]. Contrary to other E3/DUB pairs where the DUB completely deubiquitinates its E3 or a substrate, ataxin-3 instead appears to control ubiquitin chain length on CHIP substrates and deubiquitinates CHIP once polyubiquitinated substrates have assembled [132], which is consistent with ataxin-3 preferentially cleaving chains comprised of at least four ubiquitin moieties [88]. CHIP levels are unaltered in ataxin-3 knockout mice, which suggests that when CHIP and ataxin-3 normally interact [133], this modulates CHIP activity within the ubiquitinating complex rather than mediating CHIP degradation [132]. However, polyQ-expanded ataxin-3 binds CHIP with a 6-fold greater affinity compared with its wild-type counterpart [132]. By an unknown mechanism, this interaction triggers degradation of CHIP, as confirmed by the finding of reduced CHIP levels in the brains of transgenic MJD mice [132]. It is well established that ataxin-3 promotes proteasomal degradation of substrates [11,97], however this opposes the typical role of DUBs in deubiquitinating and therefore rescuing substrates from proteasomal degradation [78]. As such, it remains to be determined whether targeted degradation of CHIP by polyQ-expanded ataxin-3 is a gain-of-DUB-function effect, or rather it is the case that the increased binding between polyQ-expanded ataxin-3 and CHIP interferes with normal functioning of CHIP and entangles CHIP in degradation [134].

Although ataxin-3 is expressed throughout all regions of the brain [45,152], intranuclear inclusions are particularly abundant within the pontine neurons but are also found in the other brainstem neuronal populations, as well as the thalamus and substantia nigra [30]. Despite the fact that inclusions are a pathological hallmark of MJD, and high expression of full-length ataxin-3 causes cell death and formation of intranuclear inclusions, there is no clear correlation between the presence of intranuclear inclusions and cell death [153]. A recent study demonstrated region-specific changes in the abundance of ubiquitinated proteins in MJD mice, with expression of polyQ-expanded ataxin-3 resulting in significantly increased abundance of K63-ubiquitinated proteins in the cerebellum and brainstem, and decreased abundance of K48-ubiquitinated proteins in the brainstem of 7-week-old MJD mice [100]. It would therefore be interesting to further explore whether changes in the DUB function of ataxin-3 due to polyQ expansion affects some cell types more than others, and whether this correlates with the cell types that are particularly impacted in MJD.

## Does the DUB function of ataxin-3 have a neuroprotective role?

PolyQ expansion diseases such as MJD have historically been characterised as dominant disorders in which the mutant allele causes disease while the wild-type allele is thought to have no impact on the phenotype or progression of the disease [33,154]. It is therefore intriguing that individuals carrying two mutant *ATXN3* alleles (homozygotes) experience earlier onset of disease [155–158], as this may suggest a potential loss of the important protective function of wild-type ataxin-3. Alternatively, earlier onset in homozygotes may simply be due to the stronger effect of two mutant *ATXN3* alleles compared with just one mutant allele (heterozygotes), which is consistent with homozygous MJD mice displaying more pronounced impairment than heterozygous MJD mice [159,160]. It is also interesting that across the polyQ expansion diseases, a CAG repeat length of 35–40 is typically sufficient to cause disease, however this threshold is considerably higher in MJD and spinocerebellar ataxia type 17 [161]. This raises two questions: Does wild-type ataxin-3 influence the pathogenesis of MJD? And is wild-type ataxin-3 somehow neuroprotective?

**Figure 3.**
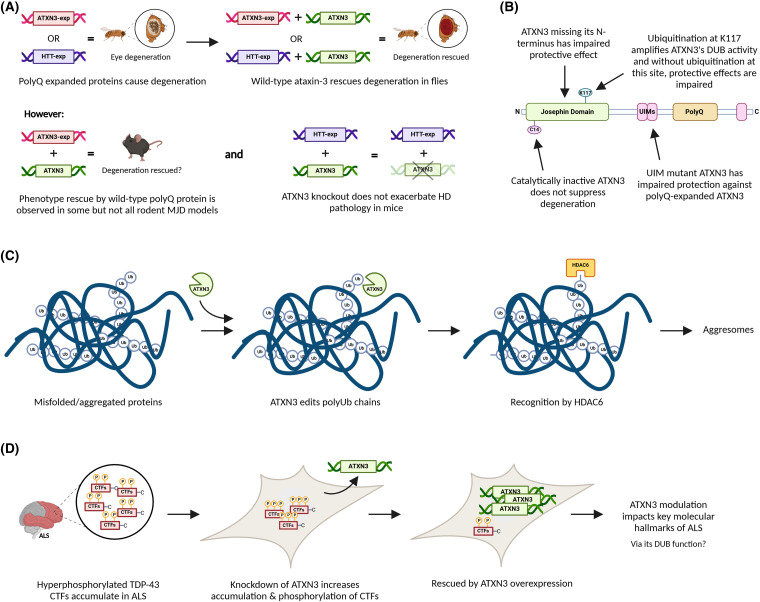
Summary of proposed neuroprotective functions of wild-type ataxin-3 protein. (**A**) Wild-type ataxin-3 appears to suppress degeneration induced by pathogenic polyQ proteins in *Drosophila* [121,162,165] and some mouse models [159], but not in other rodent models [166–168]. (**B**) The protective effects of wild-type ataxin-3 require its DUB functions. (**C**) Wild-type ataxin-3 cleaves polyubiquitin chains on misfolded/aggregated proteins, allowing them to be recognised by HDAC6 and therefore sequestered to aggresomes [96,169]. (**D**) Wild-type ataxin-3 has been implicated in modulating C-terminal TDP-43 fragments, a molecular hallmark of ALS [170]. ATXN3-exp denotes polyQ-expanded ataxin-3, HTT-exp denotes polyQ-expanded huntingtin, CTF is C-terminal TDP-43 fragment. Figure created with BioRender.com.

It is possible that the longer repeat threshold observed in MJD is due to a combination of wild-type ataxin-3 being neuroprotective, which is counteracted by the polyQ toxicity, and pathogenic ataxin-3 conserving some capacity to attenuate its own toxicity [162]. This could also explain the earlier onset and enhanced disease severity observed in homozygous MJD patients — they carry two disease-causing *ATXN3* alleles and lack the protective function carried by wild-type ataxin-3. Wild-type huntingtin (htt) protein, the toxic polyQ protein implicated in Huntington's disease, has been demonstrated in cell culture and *in vivo* models to be capable of neutralising the toxicity of polyQ-expanded htt [163,164]. Exogenous expression of ataxin-3 protein in *Drosophila melanogaster* has been shown to be protective against toxic polyQ-expanded proteins including ataxin-3, ataxin-1, and htt [121,162,165] (summarised in Figure 3). Co-expression of wild-type ataxin-3 and pathogenic polyQ-expanded ataxin-3 restored the degeneration of *Drosophila* eyes that is observed with expression of polyQ-expanded ataxin-3 alone, and flies co-expressing both forms of ataxin-3 lived longer than flies expressing only the pathogenic form [162]. These findings suggest that ataxin-3 is capable of mitigating toxicity caused by polyQ expansion. Similar findings were reported by Tsou et al. [121]. In a *Drosophila* model of Huntington's disease, co-expression of wild-type ataxin-3 and polyQ-expanded htt resulted in complete restoration of the neurodegeneration that is typically observed with expression of pathogenic htt alone [162]. Wild-type ataxin-3 serving a protective role and reducing toxicity caused by the pathogenic form of the protein has also been proposed in mice [159]. Furthermore, while exogenous expression of wild-type ataxin-3 has the effect of dramatically suppressing polyQ-expanded ataxin-3-induced degeneration in *Drosophila* [121,162], catalytic site mutant (C14A) ataxin-3 does not appear to suppress degeneration [121], suggesting that the DUB function of ataxin-3 is pivotal to its protective role.

Evidence surrounding the potential protective function of ataxin-3 protein is, however, somewhat conflicting and remains to be clarified. Knockout of the *Atxn3* gene in mice expressing pathogenic polyQ-expanded htt was not found to exacerbate polyQ protein accumulation or abundance of inclusions, suggesting that ataxin-3 may not be markedly neuroprotective in Huntington's disease [166]. In addition, contrary to studies reporting wild-type ataxin-3 to be markedly neuroprotective against polyQ-expanded ataxin-3 in *Drosophila* [162], no rescue in disease phenotype, nuclear inclusion formation, or premature death was observed for double transgenic mice co-expressing wild-type and polyQ-expanded ataxin-3 compared with mice expressing polyQ-expanded ataxin-3 alone [167]. In the study by Hübener et al. [167], wild-type ataxin-3 did not appear to markedly suppress toxicity caused by polyQ-expanded ataxin-3, and this is supported by a study in a rat model of MJD [168].

Ataxin-3 itself is ubiquitinated at the K117 residue, which dramatically amplifies its DUB activity [90,103]. Tsou et al. [121] demonstrated that ubiquitination of ataxin-3 at K117 is critical to ataxin-3's protective role against polyQ-mediated degeneration in *Drosophila*. Ataxin-3 with all lysine residues mutated to arginine (preventing lysine ubiquitination) appears to suppress eye degeneration in flies expressing polyQ-expanded ataxin-3 early in life, however, by two weeks of age this suppression is substantially lower than that observed for flies expressing wild-type or ubiquitinatable ataxin-3 [121]. Degeneration is also more severe in flies co-expressing polyQ-expanded ataxin-3 and ataxin-3 with all lysine residues mutated to arginine, compared with those co-expressing polyQ-expanded and ubiquitinatable ataxin-3 [121]. The observation of minimal intranuclear inclusions in flies co-expressing polyQ-expanded and wild-type ataxin-3 [121] indicates that perhaps ataxin-3 is protective by suppressing the formation of inclusions rather than altering ataxin-3 protein levels, and ataxin-3's DUB role may be central to this. Consistent with these findings, a recent study reported that polyQ-expanded ataxin-3 with the K117 residue mutated to prevent ubiquitination at the site, is more toxic than polyQ-expanded ataxin-3 with an intact K117 residue in *Drosophila*, further suggesting that ubiquitination at K117 is protective in MJD [171].

Further evidence in support of ataxin-3's DUB role being involved in its protective function arise from studies identifying that UIM-mutant ataxin-3 has an impaired capacity to suppress polyQ-mediated degeneration of *Drosophila* eyes [162], implicating the UIMs as critical to ataxin-3's protective function. Similarly, the N-terminus of ataxin-3, containing the DUB Josephin domain, also appears to be central to ataxin-3's protective function. When pathogenic ataxin-3 and wild-type ataxin-3 containing the C-terminal region (without the N-terminal region) were co-expressed in *Drosophila*, severe eye degeneration was not significantly suppressed [162]. However, co-expression of pathogenic ataxin-3 and ataxin-3 containing the N-terminal region, but lacking the polyQ domain, resulted in suppression of polyQ-mediated degeneration [162]. These results are indicative of the N-terminal region being critically involved in ataxin-3's protective function against polyQ-mediated degeneration, and additionally demonstrates that the polyQ region itself is not required for this activity.

Ataxin-3's role as a DUB enzyme may also serve an important neuroprotective function in other neurodegenerative diseases, such as ALS. One mechanism by which cells handle un-degraded and aggregated proteins is through the formation of aggresomes [172]. Aggresomes are dynamic structures capable of recruiting chaperones and proteasomes to facilitate degradation of aggregated proteins [173,174], and they are therefore considered to reflect cellular attempts at protection [175]. For misfolded proteins to be transported to aggresomes, they must be recognised by histone deacetylase 6 (HDAC6), which recognises and binds to the C-termini of unanchored polyubiquitin chains within aggregates [169]. It has been identified that via its DUB activity, ataxin-3 cleaves misfolded/aggregated proteins in this way to expose these motifs for recognition by HDAC6, which therefore implicates ataxin-3 in aggresome formation [96,169] (summarised in Figure 3). Mutations in the Cu/Zn superoxide dismutase (*SOD1*) gene are causative for approximately one fifth of familial ALS cases [176], and mutant SOD1 proteins are known to form aggresomes [172,176,177]. Under normal conditions, misfolded SOD1 is ubiquitinated and degraded by the proteasome in a manner presumably relying on K48-linked polyubiquitin chains [54,178,179]. However, when the UPS is overworked, K63-linked ubiquitination appears to direct misfolded proteins to aggresomes, and as a DUB, ataxin-3 seems to promote this process by editing K63-linked polyubiquitin chains on misfolded proteins to the exact length needed for the proteins to interact with constituents of the aggresome pathway [180]. By this mechanism, it appears that ataxin-3 may serve a neuroprotective role in mutant-SOD1 induced ALS, by promoting sequestration and subsequent degradation of misfolded SOD1 proteins. Interestingly, ataxin-3 has also been found to localise to and regulate the formation of aggresomes sequestering misfolded proteins involved in other forms of neurodegeneration [180–184], and this process requires the catalytic domain and UIMs of ataxin-3 [96]. One interpretation of this is that ataxin-3 contributes to pathology in other neurodegenerative diseases. Alternatively, because such inclusions are thought to reflect cellular attempts at protection, this may suggest that ataxin-3's normal function is involved in dealing with aberrant, aggregated, and neurotoxic proteins in these neurodegenerative diseases in a protective manner [162].

In addition to interacting with the ALS-linked SOD1 protein, ataxin-3 also interacts with another ALS-linked protein, VCP/p97, as discussed [11]. PolyQ expansion in ataxin-3 has not been associated with ALS risk, however a recent 3′untranslated region (3′UTR) alternative polyadenylation transcriptome-wide association study (3′aTWAS) discovered *ATXN3* to be a 3′TWAS-significant gene for ALS [170]. TDP-43 C-terminal fragments are observed in the post-mortem tissue of 97% of ALS patients [185,186], and pathological TDP-43 inclusions are similarly observed in lower motor neurons in the brainstem and spinal cord of MJD patients [187]. The study by Cui et al. [170] subsequently showed that knockdown of *ATXN3* resulted in a substantial increase in the accumulation and phosphorylation of TDP-43 C-terminal fragments, and that overexpression of *ATXN3* reduced this phenotype, thereby demonstrating that *ATXN3* modulation impacts important molecular hallmarks of ALS pathology *in vitro*. Whilst Cui et al., did not investigate the mechanism underpinning this effect of *ATXN3* on phosphorylated C-terminal fragments of TDP-43, it is plausible that the DUB function of ataxin-3 may be involved (Figure 3).

Increased ubiquitination of polyQ-expanded ataxin-3 has been observed in an MJD mouse model [90], suggesting a link between ubiquitination of polyQ-expanded ataxin-3 and MJD pathogenesis. Since polyQ-expanded ataxin-3 retains its neuroprotective capacity in *Drosophila* [162] and ubiquitination of ataxin-3 enhances its activity, Todi et al. [90] propose that ubiquitination of polyQ-expanded ataxin-3 may serve a neuroprotective function in MJD mice and increased catalytic activity would enhance this neuroprotective function [103]. In other words, it is possible that ubiquitination of polyQ-expanded ataxin-3 culminates in a gain-of-function effect whereby the DUB activity is more active and enhances suppression of its own neurotoxicity, although this too remains to be clarified.

## The DUB function of ataxin-3 regulates transcription

Ataxin-3 also functions as a regulator of transcriptional activity [13,14,18], and is known to interact with several transcription factors including CREB-binding protein (CBP) [13,188,189], p300 [13], p300/CBP-associated factor (PCAF) [13], HDAC3 and HDAC6 [14,96], and others [190,191]. In addition, many of the known substrates of ataxin-3's DUB function (listed in Table 1) are involved in transcription, including CBP, HIF1α, KLF4, YAP, and HDAC3. It is therefore intriguing to consider the intersection between ataxin-3's role as a DUB enzyme and regulator of transcription.

The human ataxin-3 protein is predicted to contain a nuclear localisation signal between residues 273–286 [192–195], which lies within the ubiquitin-binding domain and near the polyQ region. Ataxin-3 is therefore capable of being transported to the nucleus [192], and appears to bind DNA directly via a leucine zipper motif between amino acids 223–270 [14], which binds a consensus DNA sequence [196]. In two *Caenorhabditis elegans* models, knockdown of the *ATXN3* ortholog resulted in differential expression of 290 genes, with up-regulation of 253 genes and down-regulation of 37 genes compared with wild-type control animals [7]. Taken together, this provides evidence supporting a role for ataxin-3 in transcription regulation. Furthermore, the SCF (skp-1, cullin, F-box) complex is an E3 ubiquitin ligase that targets substrates, including transcription factors, to proteasomal degradation. Ataxin-3 has been identified as a putatative genetic interactor with several SCF-associated genes [7], and the *ATXN3* knockout strains had several down-regulated SCF complex genes [7], suggesting an indirect, upstream mechanism of transcriptional regulation for ataxin-3.

**Figure 4.**
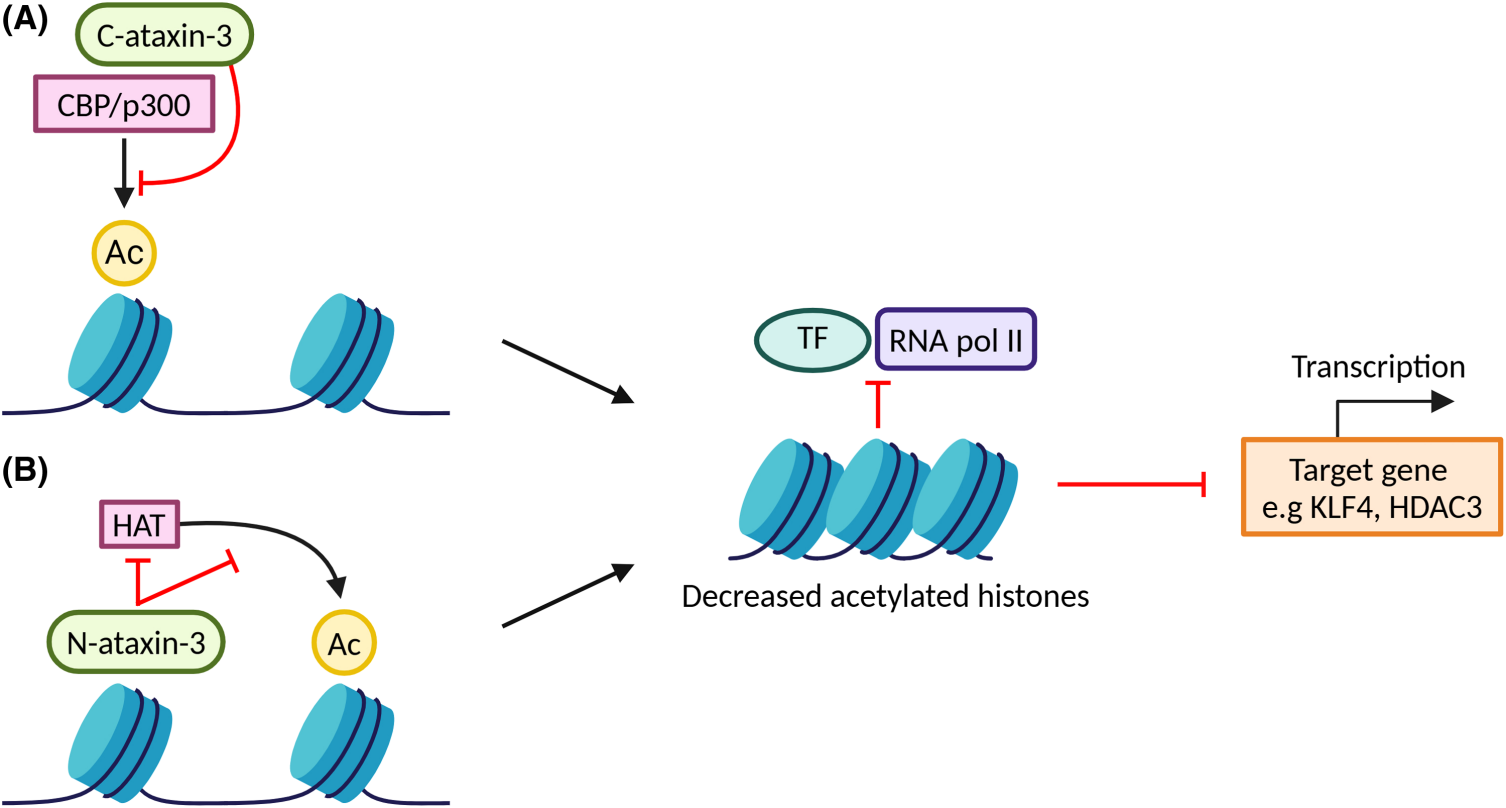
Ataxin-3 can repress transcription via at least two mechanisms. (**A**) The C-terminal region of the ataxin-3 protein (C-ataxin-3) binds to CREB-binding protein (CBP) and p300 co-activators and represses their transcriptional activity, preventing acetylation of histones, and resulting in chromatin being more tightly packed and made inaccessible to transcription factors and RNA pol II. As a result, transcription of the target gene is repressed. (**B**) The N-terminal region of ataxin-3 protein (N-ataxin-3) binds to histones and prevents their acetylation by HATs, which also results in repression of transcription by a similar mechanism as (**A**). TF is transcription factor, Ac is acetyl group, RNA pol II is RNA polymerase II, HAT is histone acetyltransferase. Figure created with BioRender.com.

Ataxin-3 appears to directly repress transcription by at least two mechanisms — via its N-, and C-termini [13] (Figure 4). Via its polyQ-containing C-terminus, ataxin-3 binds to and directly represses the transcriptional activity of CBP and p300 co-activators, and PCAF [13]. Binding between ataxin-3's C-terminus and co-activators is stronger when the polyQ region is expanded, however it is unclear whether it is solely the polyQ repeat region that is responsible for binding co-activators to repress transcription, or whether other residues in the C-terminus also play a role in this process [13]. Via its N-terminus, ataxin-3 behaves as an inhibitor of histone acetyltransferases subunit by binding to histones and preventing their acetylation by HATs [13]. Acetylation of histones is the main epigenetic change altering the accessibility of genes to transcription factors by manipulating how tightly nucleosomes are packed [197]. These acetyl groups decrease the affinity of DNA for histones, resulting in relaxation of tightly packed chromatin, thereby making it accessible to transcription factors and RNA polymerase II (RNA pol II). Thus, by preventing the acetylation of histones, ataxin-3 prevents transcription factors and RNA pol II from accessing DNA, resulting in transcription repression.

In addition to these mechanisms of transcription regulation, ataxin-3 is also hypothesised to regulate transcription via its DUB activity by impacting the turnover of transcription factors and therefore manipulating the formation and activity of repressor complexes [7,14]. This proposed function is consistent with other DUB enzymes that remove ubiquitin from substrates that are responsible for regulating transcription [198,199].

One example of the DUB function of ataxin-3 regulating gene transcription is evident in ataxin-3's interaction with Krüppel-like factor 4 (KLF4). KLF4 is a transcription factor that acts as a tumour suppressor in some cancers, and as an oncogene in other cancers [200]. Ataxin-3 and KLF4 interact via amino acids 220–240 of KLF4 and amino acids 1–205 of ataxin-3 which contains the DUB Josephin domain [21]. Ataxin-3 has been shown to deubiquitinate and therefore stabilise KLF4, and it is hypothesised that the up-regulation of *ATXN3* observed in breast cancer contributes to up-regulation of KLF4, thereby accelerating breast cancer metastasis [21]. However, the mechansims of *ATXN3* up-regulation in breast cancer, how KLF4 promotes breast cancer, and whether aberrant deubiquitination of KLF4 by ataxin-3 is involved in the pathophysiology of MJD, remain to be explored.

Another example of ataxin-3's capacity to regulate gene expression via its DUB function is evident in its interaction with histone deacetylase 3 (HDAC3). HDAC3 is degraded by the UPS [201–203], and protein levels of HDAC3 appear to be tightly regulated by ataxin-3 as knockout of endogenous *ATXN3* robustly down-regulates HDAC3, while overexpession of *ATXN3* significantly up-regulates HDAC3 [18]. Ataxin-3 is also known to deubiquitinate both K48- and K63-linked ubiquitin chains on HDAC3 in a Josephin domain-dependent manner, resulting in stabilisation and increased protein levels of HDAC3 [18]. The interaction between wild-type ataxin-3 and HDAC3 is associated with transcription repression because ataxin-3 increases the deacetylase activity of HDAC3 complexes and increases deacetylation of histone H3 [14]. PolyQ-expanded ataxin-3 reportedly loses its repressor function and gains a transcription activator function, resulting in reduced deacetylase activity of HDAC3 complexes and increased acetylation of histone H3 [14]. It is hypothesised that this phenomenon may in part explain the altered gene expression observed in MJD [204,205]. A recent study has shown that ataxin-3 and HDAC6 co-immunoprecipitate and interact [206], as suggested by a previous surface plasmon resonance study [207], and overexpression of ataxin-3 promotes the deubiquitination of HDAC6 in pancreatic cancer cells [206]. The resulting up-regulation of HDAC6 was found to repress expression of E-cadherin by deacetylation in the promotor region of E-cadherin [206]. Whether ataxin-3 directly deubiquitinates HDAC6 and the potential impact of polyQ-expansion on its capacity to do so remains to be investigated, however, these studies suggest a possibility of ataxin-3 regulating the transcription functions of HDAC6 via its DUB function.

## Conclusions

Although the single genetic cause of MJD has now been known for over three decades, MJD remains a devastating and fatal neurodegenerative disease. In this review, we aimed to consolidate the literature on ataxin-3's function as a DUB enzyme to aid our understanding of this important function.

The literature demonstrates that the deubiquitinase function of ataxin-3 is important because it can regulate abundance and activity of various proteins, and likely has a range of protective effects for the cell. As we continue to unravel ataxin-3's function as a DUB enzyme, it is likely that we will find more examples of ataxin-3 regulating more than just the proteostasis of proteins via its DUB function, including other examples where ataxin-3's DUB function regulates transcription. In gaining a greater understanding of these impacts, we will hopefully identify novel therapeutic targets for the treatment of MJD.

Previous studies have also suggested that the DUB function of ataxin-3 may be involved in the pathophysiology of other diseases in addition to MJD, and it also appears to be the case that ataxin-3 may have some protective effects for these diseases. As such, inroads made in relation to understanding the DUB function of ataxin-3 in MJD will likely also be important for understanding the pathophysiology of other diseases including neurodegenerative diseases and cancer.

## Open Access

Open access for this article was enabled by the participation of Macquarie University in an all-inclusive *Read & Publish* agreement with Portland Press and the Biochemical Society under a transformative agreement with CAUL.

## Abbreviations

ALS: amyotrophic lateral sclerosis
CAG: cytosine-adenine-guanine
CBP: CREB-binding protein
DUB: deubiquitinating
ERAD: endoplasmic reticulum-associated protein degradation
HDAC3: histone deacetylase 3
HDAC6: histone deacetylase 6
htt: huntingtin
KLF4: Krüppel-like factor 4
MJD: Machado-Joseph disease
NLS: nuclear localisation signal
PCAF: p300/CBP-associated factor
SOD1: superoxide dismutase
UIM: ubiquitin-interacting motif
UPS: ubiquitin-proteasome system (UPS)
VCP: Valosin-containing protein
